# Antidepressant- and Anxiolytic-like Effects of Pomegranate: Is It Acting by Common or Well-Known Mechanisms of Action?

**DOI:** 10.3390/plants13162205

**Published:** 2024-08-09

**Authors:** Erika Estrada-Camerena, Carolina López-Rubalcava, Nelly Maritza Vega-Rivera, María Eva González-Trujano

**Affiliations:** 1 Laboratorio de Neuropsicofarmacología, Dirección de Neurociencias, Instituto Nacional de Psiquiatría “Ramón de la Fuente Muñiz”, Mexico City 14370, Mexico; nmvega@inprf.gob.mx; 2 Laboratorio 17, Departamento de Farmacobiología, Centro de Investigación y Estudios Avanzados, Sede Sur, Mexico City 14330, Mexico; clopezr@cinvestav.mx; 3 Laboratorio de Neurofarmacología de Productos Naturales, Dirección de Neurociencias, Instituto Nacional de Psiquiatría “Ramón de la Fuente Muñiz”, Mexico City 14370, Mexico; evag@inprf.gob.mx

**Keywords:** anxiety, depression, estrogen receptors, serotonin, oxidative stress, peroxisome proliferator-activated receptor gamma, perimenopause, pomegranate

## Abstract

The pharmacological effects of pomegranates have been described considering metabolic aspects such as hypoglycemic and hypolipidemic activities. The pomegranate extract has activity on the central nervous system (CNS) as a natural antidepressant and anxiolytic. The chemical composition of pomegranates is complex since the bioactive compounds are multiple secondary metabolites that have been identified in the extracts derived from the peel, seed, flowers, leaves, or in their combination; so, it has not been easy to identify an individual compound as responsible for its observed pharmacological properties. From this point of view, the present review analyzes the effects of crude extracts or fractions of pomegranates and their possible mechanisms of action concerning antidepressant- and anxiolytic-like effects in animal models. Serotonin receptors, estrogen receptors, the peroxisome proliferator-activated receptor gamma (PPARγ), or monoamine oxidase enzymes, as well as potent antioxidant and neuroplasticity properties, have been described as possible mediators involved in the antidepressant- and anxiolytic-like behaviors after pomegranate treatment. The pharmacological effects observed on the CNS in experimental models associated with a specific stress level suggest that pomegranates could simultaneously modulate the stress response by activating several targets. For the present review, scientific evidence was gathered to integrate it and suggest a possible pathway for mediators to be involved in the mechanisms of action of the pomegranate’s antidepressant- and anxiolytic-like effects. Furthermore, the potential benefits are discussed on comorbid conditions with anxiety and depression, such as perimenopause transition and pain.

## 1. General Description: Botanical Aspects and Bioactive Compounds of Pomegranates

*Punica granatum* L. (Lythraceae) or pomegranate belongs to the *Punicaceae* family. It is a deciduous shrub or a small tree with perennial rootstock [1], that can grow up to 12 to 16 feet and live over 200 years. The leaves are glossy, and the flowers are red, white, large, or variegated and have tubular calyxes. Pomegranate fruit is grenade-shaped with a deep red, leathery skin and a crown-shaped calyx. The seeds are surrounded by a small amount of tart and red juice and are separated via a white, membranous pericarp [2].

Generally, pomegranates are categorized as ornamental, having white flowers, small fruits, and hard seeds, and edible pomegranates with medicinal value are categorized as having red flowers, large fruits, and soft seeds [3]. The edible part of pomegranates is about 57% to 85% of the whole fruit, of which the fruit juice represents 36% to 63% [3].

Over the last decades, the pomegranate and its extracts have demonstrated several beneficial health properties, which has led it to be considered a functional food. Clinical and preclinical studies have shown that pomegranates have antioxidant [4,5], anti-inflammatory, anti-tumorigenic, anti-microbial [5], anti-obesity [5,6,7], anti-nociceptive [8], neuroprotective [9], and more recently anxiolytic- and antidepressant-like properties [7,10,11,12]. Interestingly, most of the health benefits of pomegranates are attributed to its high content of polyphenols, which represents 26–30% of the total weight of the fruit [5,8].

Pomegranate contains 17 types of amino acids and an array of minerals, especially a high content of vitamin C, calcium, iron, and phosphorus, as well as a small amount of retinol, riboflavin, ferulic acid, and other phenolic compounds that are nutritious and beneficial to human health [13,14]. Table 1 shows the main phytochemicals reported for pomegranates. Phenolic compounds include polyphenols, anthocyanidins, catechins, flavanols, and isoflavones [8,15,16,17,18,19,20,21]; some are phytoestrogens.

**Figure 1:**
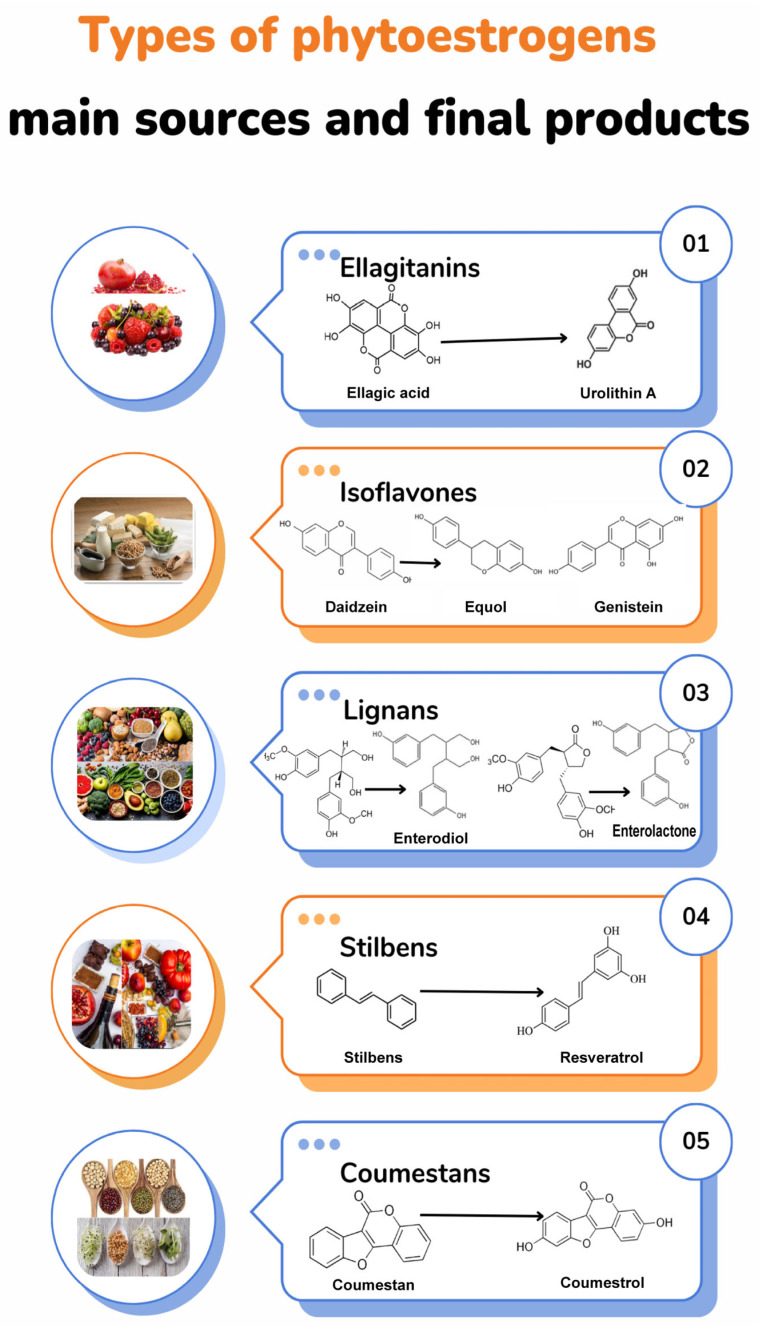
Schematic representation of phytoestrogen classification. Pomegranate and berries (strawberries, raspberries, blueberries, and blackberries) and nuts (walnuts, pecans, and chestnuts) are primary sources of ellagitannins, which are biotransformed in urolithins by microbiota (1); soy-derived products, groundnut, and tea are the primary source of isoflavones, which are finally converted to genistein and daidzein and, finally, to equol after a biotransformation process (2). Lignans (matairesinol and secoisolariciresinol) come from cereals (barley, oats, wheat, and corn), vegetables (curly kale, broccoli, asparagus, carrots, and garlic), fruits (apricot, strawberry, peach, and orange), and nuts (flaxseed, sesame, sunflower, and cashew) and are biotransformed to enterolactone and enterodiol (3). Stilbens represented by resveratrol are found in wine, peanut, red grapes, cocoa, pistachios, and blueberries (4). Coumestans, represented by coumestrol, can be found in legumes, sprouts of soy, alfalfa, Brussels sprouts, clover, and chickpeas (5). Stilbens and coumestans production appears to be independent of microbiota [16,27,28].

**Figure 2:**
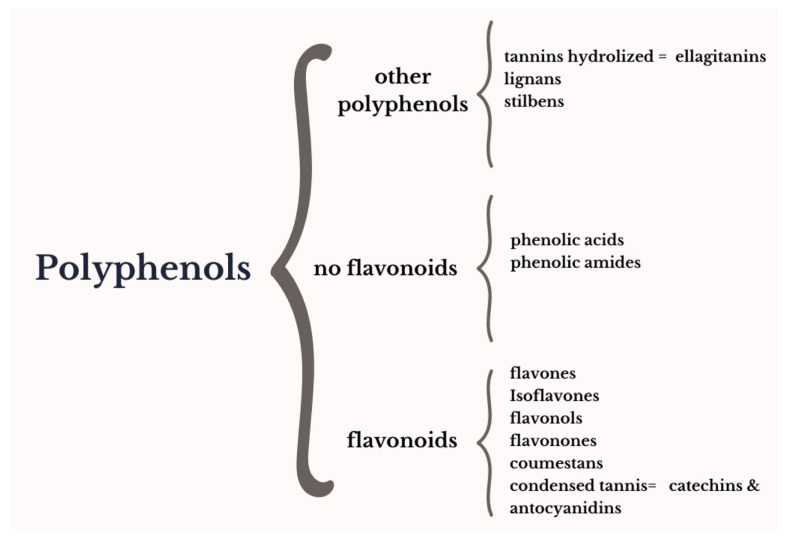
Schematic representation of the most representative groups of polyphenols classified according to their chemical structure in flavonoids (flavan nucleus), no flavonoids (phenolic amides and acids), and other phenolic groups (lignans, stilbenes, and hydrolyzed tannins) [21,22,29,30].

Phytoestrogens are molecules derived from plant metabolism with a chemical structure similar to 17β-estradiol and show activity on estrogen receptors (Figure 1). The phytoestrogens more recognized are isoflavones (daidzein, genistein, and equol), stilbenes (resveratrol), ellagitannins (urolithin), lignans (enterolactone and enterodiol), and coumestans (coumestrol) (Figure 1) [22]. However, other compounds have been identified showing activity on estrogen receptors (ERs) and activity on the CNS [23,24,25]; for example, the condensed tannin, anthocyanins, flavonols, such as quercetin and kaempferol (Figure 2), and luteolin [15,17,19,20]. From these data, it has been proposed that pomegranates are a functional food rich in phytoestrogens. Under this premise, some research groups have sought its therapeutic potential for the treatment of menopause symptoms.

**Table 1:** Phytochemicals reported in pomegranates and having activity on estrogen receptors.

Phytochemical	Phytoestrogen Group	Metabolite	Estrogen Receptor Activity	References
*Isoflavones*	Genistein		ERa	[5,20]
	Daidzein		ER memb	
	Equol		ERb	
*Flavonols*	Quercetin	Rutin (Quercetin-3-O-rutinoside)	ERa/b	[15,17,20]
	Kaempferol		ER memb ERb	
	Myricetin		Era	
*Condensed tannins*	Anthocyanidins	Cyanidin-3,5 di-O- diglucoside Cyanidin-3-O-glucoside Delphinidin 3,5 di-O- diglucoside Delphinidin 3-O-glucoside Pelargonidin-3,5-di-O-diglucoside Pelargonidin-3-O-glucoside	ERa/b ERa ER memb	[8,15,17,18,20]
	Catechins	Epicatechin Epicatechin galleate Chysin	ERa, GPR30 ER GPR30 ERa	
*Coumestans*	Coumestrol		ERa/b ER memb	[5]
*Flavonons*	Luteolin Apigenin		ERa/b ERa/b	[19]
*Lignans*	Isolariciresinol Matairesinol		ERa/b	[20]
*Stilbenes*	Resveratrol		ND	
*Flavanones*	Baicalein Naringin		ER memb ERa, ER memb	[26]
*Hydrolyzed tannins*	Ellagitannins	Punicalagin α and β	ERb	[8,15,17,21]
		Ellagic acid glucoside Ellagic acid	ND	
*Phenolic acids*	Ferulic acid Cinnamic acid Coumaric acid Gallic acid Caffeic acid		ERa	[13,17,20]

Data from the literature using extracts of pulp, juice, peel, seed, or the whole fruit, obtained via high-performance liquid chromatography combined with different methods and their activity on estrogen receptors (ERs) α, β, or membrane estrogen receptor (GPR30 or ERmemb).

## 2. Menopause Is a Natural Process Altering the Endocrine System

Menopause is a natural process in women’s lives, indicative of endocrine aging in which menstrual cycles disappear, stopping the reproductive period. Hormonally, menopause consists of a permanent decline of 17-β estradiol (E2) and progesterone peripheral levels with the concomitant increase in gonadotrophins, which are follicle-stimulating and luteinizing hormones. Menopause transition, named perimenopause, initiates several months before the last menstruation and is characterized by irregular hormonal fluctuations of estrogens, progestins, and gonadotrophins (FSH and LH), which determine irregular cycles. The hormonal milieu that prevails in this period impacts several levels in the organism, including the brain areas related to the control of emotions, such as the amygdala, hippocampus, and prefrontal cortex through specific hormone receptors such as estrogen receptors α and β) [31] and protein-G coupled receptor-1 (GPER-1). 

During perimenopause and menopause, some women experience symptoms that affect their quality of life, such as anxiety, depression, an increase in stress perception, hot flushes, insomnia, and, in some cases, metabolic alterations that have been partially associated with estrogen oscillations [32,33,34]. In a small but well-designed study, Gordon et al. [35] showed that a more significant fluctuation of estrone-3-glucuronide, a urine metabolite of estradiol, from one week to the next, was negatively associated with negative affect, anhedonic depressive symptoms, higher heart rate, diastolic blood pressure, rejection, anger, and sadness during stressful situations [35]. The authors postulated that more significant estrogen fluctuations increase stress sensitivity to psychosocial stressors and may predispose to the development of perimenopausal depression [33,35]. Further, when low and stable estrogen levels were observed, a decreased risk of depressed mood was also observed [35,36,37]. The mechanism via which estrogens modulate the negative mood behavior during the perimenopause transition is unknown. However, some evidence suggests the role of a previous history of depression and functional alterations of the hypothalamus–pituitary–adrenal glandule axis [35]. Following this line of evidence in post-mortem studies, other estrogen receptors showed lower expressions of ERα in the prefrontal cortex and hippocampus of women with major depression [38]. Moreover, Iqbal et al. [31] reported in a review that ERα and β were involved in negative emotional memories; ERα, ERβ, and GPER via inflammation and oxidative pathways in bipolar patients; ERα, ERβ, BDNF, and TRkB pathways in major depression; and ERα, ERβ, GPER, and the GPER- and MAPK signaling pathways in anxiety disorders (generalized anxiety and post-traumatic stress disorder) [31,39]. Therefore, based on this information, it is difficult to establish a conclusion about the role of estrogen receptors in a specific pathology. 

In contrast, basic research that has explored the role of E2 and phytoestrogens and their receptors suggests that ERB mediates the regulation of anxiety- and depressive-like behaviors in animal models in specific brain areas such as the hippocampus and amygdala [22,40,41,42,43,44,45], whereas ERα could mainly mediate peripheral effects such as in the uterus and mammary glands, among others [46]. Several animal models using chronic and acute stress for depressive-like behavior, elevated plus-maze and open-field tests for anxiety-like behavior, specific ER antagonists, as well as knockout mice have shown consistent evidence that ERβ and GPR30 are involved in the modulation of depressive and anxiety conditions through estrogen-like compounds [44,47,48].

On the other hand, estrogen replacement therapy (ERT) showed, in some populations, a positive effect on mood disorders, particularly the antidepressant effect; improved the quality of life of women; and contributed to preventing dyslipidemia and hypertension [49,50,51]. Based on this evidence, several groups have explored natural products containing phytoestrogens, which, when used as ERT, could be safer than estradiol [52]. These phytochemicals act as selective estrogen receptor modulators (SERMs), i.e., they could be agonists or antagonists depending on the receptor and its location. For example, some SERMs act as agonists on ERs located in the brain and as antagonists in the uterus and mammary gland [53,54], so their use could represent an advantage.

Thus, in 2012, the therapeutic effect of a 12-week pomegranate seed oil (PGS) treatment for menopausal symptoms was investigated. Although the PGS reduced such symptoms, it did not achieve significance. However, the authors remarked on the importance of evaluating the PGS for a more extended period [55]. Also, a systematic review reported the effect of pomegranate juice on osteoporosis, osteoarthritis, or rheumatoid arthritis, reporting positive effects [56].

Nevertheless, other symptoms related to mood alterations, frequently present in perimenopause, have been little studied (Table 2). Few clinical trials have explored the effect of pomegranate consumption on anxiety, depression, or stress perception in perimenopause, always being considered as a secondary outcome of studies exploring other symptoms of menopause (Table 2). A recent meta-analysis and systematic review indicate that pomegranates are helpful for hot flushes and sleep disturbances, according to a menopause rating scale [49]. Furthermore, the studies include the combination of pomegranates with other plants or phytonutrients, making it difficult to discern whether or not the effects are due to pomegranate intake. Therefore, it is necessary to provide more information derived from randomized placebo-controlled studies that evaluate the anxiolytic and antidepressant potential of pomegranates.

The present review is focused on analyzing the effect of pomegranates on depression- and anxiety-like behavior, two conditions associated with perimenopause, exploring the possibility of a common mechanism of action based on preclinical studies. Only those studies in which pomegranate extract was used are included. Information was selected according to the effect of pomegranates or *Punica granatum* as an antidepressant or anxiolytic alone or associated with pain in clinical trials or animal models in manuscripts published in English in PubMed or Web of Science databases. We used the following as search terms: ‘pomegranate’ or ‘*Punica granatum*’ and ‘anxiety’; ‘anxiogenic’, ‘anxiety-like’, and ‘depression’; ‘antidepressant’, ‘antidepressant-like behavior’, and ‘clinical trials’; and ‘menopause,’ menopause symptoms,’ and ‘ovariectomy’ as main outcomes.

To our knowledge, this is the first review of central nervous system diseases associated with pomegranates, such as the triad anxiety, depression, and pain, which can also become comorbid. The audience or readers interested in this work may be a population, scientific or not, interested in natural products such as Punica granatum (pomegranate) as alternative treatment for psychiatric disorders and cases of anxiety and depression, which are more commonly found in general consumption in populations with chronic pain compared to the other two psychiatric disorders. This review compiles the few manuscripts found in the literature whose results give evidence of beneficial effects and a lack of adverse effects for the pharmacological activity of *Punica granatum* in contrast to what is already known for current therapy for this triad.

## 3. Antidepressant Effect on Animal Models

Regarding antidepressant-like activity, reports working with animal models in rodents, mainly ovariectomized mice and rats, showed promising results. In these studies, ovariectomy (OVX) was a strategy for simulating an endocrine condition like menopause, where the estrogen source (ovaries) was eliminated. Pomegranate was used to evaluate the effect of a natural product on specific behaviors that were utilized as an index of menopause symptoms. In some reports, rats or mice in an aging process were included, increasing the face validity of the used model [63,64].

An animal model frequently used for the screening of potential antidepressant drugs and therapies is the forced swimming test (FST) [65,66]. The FST detects passive or active behavioral strategies to cope with stress challenges [67,68,69]. For the present review, we considered that active strategies in the results of the FST increase in the face of stress with a concomitant decrease in passive coping strategies, i.e., an increase in swimming and a decrease in the immobility behavior.

Regarding the FST, it has been reported that pomegranate administration decreased the immobility behavior in a wide range of doses (0.1 to 100 mg/kg) and under several treatment schedules. For example, a dose of 1 mg/kg once daily for 14 days effectively reduced immobility behavior by 50% compared with the control group [11,12,70]. Also, using a schedule of three injections in 24 h was equally effective in reducing the immobility behavior (40% versus the control group) [12]. The different schedules of administration suggest a mechanism of action that may involve the activation of membranal receptors and signaling pathways that promote short-term changes.

In addition, the administration route did not influence the antidepressant-like effect of pomegranates since, given orally or intraperitoneally, a decrease in immobility behavior was observed [11,12]. This is relevant if it is considered that the proposed active compound involved in the estrogenic activity of pomegranates requires the metabolic process induced by microbiota, for example, urolithins [11,12,71,72]. Valdes-Sustaita et al. [12] suggested that in the aqueous pomegranate extract, active compounds involved in the antidepressant-like activity do not require a biotransformation process. Furthermore, aqueous pomegranate extract (AE-PG) does not require first-step metabolism to deliver active polar compounds to different tissues.

Other models involving chronic stress have demonstrated evidence of the antidepressant-like action of AE-PG, increasing convergence validation between models. For example, in the chronic mild stress model, extracts prepared from pomegranate peel (30 mg/kg) or flax seed (30 mg/kg) were tested after 50 days of treatment [73]. The authors found that each extract reduced anhedonia by increasing sucrose intake by 60% compared with a control group.

### Mechanism of Action Proposed on Specific Receptors

The behavioral profile induced by pomegranates in the FST was similar to that induced by antidepressants with activity on the serotonergic system [11,74,75], an increase in swimming behavior by decreasing immobility behavior. Furthermore, non-effective doses of AE-PG synergized with serotonergic drugs such as escitalopram, a selective serotonin reuptake inhibitor [11,70], and induced antidepressant-like activity. Regarding this, CIT or AE-PG alone at doses of 2.5 and 0.1 mg/kg, respectively, reduced the immobility behavior by 7%; however, when these doses were combined, the reduction was 73% versus the control group. In this line, evidence indicates that the depletion of serotonin neurotransmission induced by the neurotoxin 5, 7-dihydroxytryptamine, which destroys serotonergic terminals, canceling the antidepressant-like effect of AE-PG on the FST [12]. These data reinforce the hypothesis that pomegranates have active compounds that act on the serotonergic system to exert antidepressant-like activity.

Because pomegranates are rich in phytoestrogens, they may interact with estrogen receptors (ER) [17,19,24,25]. Based on this, it was evaluated whether the antidepressant-like effect of AE-PG was blocked with the antagonist of estrogen receptors, tamoxifen. This drug is a selective estrogen receptor modulator (SERM) that blocks the antidepressant-like action of AE-PG on the FST.

Interestingly, some phytoestrogens could act as SERMs [24], participating as antagonists in the uterus and mammary gland and as agonists in some brain structures. As far as we know, this idea has not been explored in in vitro or behavioral studies.

When exploring the activity of pomegranates on ER in the FST, specific antagonists to ERα and β were tested. The antagonist of ERβ, 4-[2-phenyl-5,7-bis(trifluoromethyl) pyrazolo [1,5-a]-pyrimidin-3-yl]phenol], PHTPP, entirely blocked the effect of pomegranates but not the antagonist to ERα, theophylline, 8-[(benzylthio)methyl]-(7Cl,8Cl), TPBM [12]. It is relevant to note that ERβ and SERMs are linked to the serotonergic system function [42,44,76,77]; therefore, AE-PG can modulate, directly or indirectly, the function of the serotonergic system. Specific studies could contribute to elucidating the possible serotonergic receptors and how the phytoestrogen content in the AE-PG modulates the activity of the serotonergic system. Notwithstanding this, membrane estrogen receptors such as GPR30 have not been explored. Because 17β-estradiol exerts an antidepressant-like action by its interaction with this receptor [78] and pomegranates also produce an anti-immobility effect in a short time [11], it is feasible that these receptors are also involved in the AE-PG actions.

Another mechanism of action has been explored using the chronic mild stress (CMS) model, where pomegranates showed antidepressant-like action due to a reduction in anhedonia-like behavior [73]. The authors investigated the effects of two extracts of pomegranate on monoamine oxidase (MAO) enzymes. Pomegranate peel extract reduced the increased MAO-B activity (46.17%) induced by CMS, while the flax seed extract reduced the activity of both MAO-A (38%) and B (59.27%) enzymes [73]. MAO-B mainly participates in the degradation of dopamine, whereas MAO-A metabolizes serotonin and noradrenaline [79]. Importantly, in the behavioral model used, anhedonia, a behavior related to dopamine neurotransmission [80,81], was the most important index of depressive-like behavior [81,82]. Therefore, the inhibition of MAO-B could contribute to an increase in dopamine and a reduction in anhedonia. However, activity on the dopaminergic system was not detected in the FST [4,11,70], which could be related to the differences between studies in dose and length of treatments. In addition, the effect on MAO-A could partly explain the activity of serotonin and its outcome on the FST.

This information suggests a complex mechanism of action on monoamine (5-HT, NA, and DA) regulation that could involve several targets on specific receptors not yet explored and enzymes depending on the type of extract, dose, and duration of treatments. It is essential to keep in mind that if an extract has more activity on MAO-B, pharmacological interactions with psychotropic and other types of drugs could be present, generating critical adverse events.

In this regard, the interaction among functional food and herbs with psychotropic drugs has been reported, some of them with significant adverse effects [83]. This topic has been poorly studied, and precise studies could provide more evidence for adequate management of specific combinations. As far as we know, reports about the toxic effects of pomegranates have not been documented. In many studies looking for such effects, doses in the 1–1000 mg/kg range were negative in rodents [7,8,84,85] and humans [61,62,86,87]. According to a systematic review, the side effects are gastrointestinal problems, flu-like symptoms, and urinary problems. In case-report studies, allergic reactions were the most significant reported side effect [88].

In relation to interactions with other drugs, although pomegranates modulate the activity of CYP450 3A4 and 2C9, affecting gastrointestinal and liver metabolism of some drugs [89], no clinically relevant interactions have been reported with psychotropic drugs, and this needs more exploration. For example, studies exploring midazolam’s oral and intravenous administration in healthy subjects reported no changes in pharmacokinetic parameters (clearance, elimination, and half-life time) after acute administration or five days of ingestion [90]. Also, it was explored if pomegranate ingestion for five days affected the pharmacokinetic parameters of midazolam and dapoxetine, a drug used to treat sexual dysfunction. These data indicate that pomegranates did not modify any pharmacokinetic parameter of dapoxetine and midazolam [91]. Other reports involving animal testing showed that pomegranate juice affected the absorption of buspirone without modifying the elimination rate [89].

Recently, the influence of pomegranate juice ingestion on the pharmacokinetics parameters of brexpiprazole, a drug used for schizophrenia and major depression, was evaluated in rats. Pomegranate increased the absorption without modifying the total concentration, half-time, and clearance, suggesting that the effect of pomegranates on this drug is restricted to the gastrointestinal tract [92]. In addition, combining low doses of AE-PG with low doses of citalopram produced an antidepressant-like action in the FST [12,70]. As mentioned before, individual doses did not produce a behavioral effect on the FST but, when combined, elicited an antidepressant-like activity by reducing the immobility behavior in 40% of subjects compared with the control group, without producing unspecific effects on locomotor activity [12,70]. This combination increased the number of dendritic thin spines (88% vs. control) and their complexity and increased BDNF levels (133% vs. control) and synaptophysin (300% vs. control) expression in the hippocampus [70]. From this point of view, it is argued that the combination of citalopram with the AE-PG could be a strategy for facilitating the antidepressant-like action of antidepressants in a menopause-like condition [12,70]. The antidepressant-like effect found with the combination seems to be mediated by ER and 5-HT on neuroplasticity. Escitalopram is metabolized mainly by the enzyme 2C19 and a minor contribution of 3A4 and 2D6, which did not imply a negative interaction with pomegranates. In this regard, specific experiments on pharmacokinetics are necessary.

However, it is essential to take into consideration when polypharmacy is present since the combination with other drugs could cause adverse events. An interaction warning between warfarin and pomegranate juice ingestion was reported with increased prothrombin time and international normalized ratio, with hemorrhage risk as a consequence. These data suggest that for drugs with antiplatelet activity (food, herb, or medications), the use of pomegranates requires medical supervision [93]. 

Therefore, more studies revealing possible interactions with other drugs are warranted.

From the current data, it is possible to see that different active compounds present in pomegranates, whether from the peel, flax seed, or the whole fruit, showed properties that may confer activity on distinct neurotransmitter systems.

## 4. Anxiolytic-like Action on Animal Models

Another psychiatric symptom of perimenopause is anxiety [94,95,96]. Clinical data report a high prevalence of anxiety, mainly when metabolic or cardiovascular alterations are present. For example, anxiety is observed in women with diabetes mellitus, dyslipidemia, and cardiovascular affection [97,98,99], among others. Research based on preclinical and clinical studies suggested that the decrease in estradiol levels due to menopause made the system more vulnerable and, in some cases, developed some pathologies. From this point of view, some research groups have explored the use of phytoestrogens to prevent, reduce, or alleviate the symptoms of both metabolic and anxiety conditions.

Estrada-Camarena et al. [7], demonstrated that the cafeteria diet (CAF) promoted increased body weight, triglycerides, cholesterol, glucose, and insulin resistance in OVX-aged rats. This condition resembled a condition like menopause. In this model, CAF-fed rats showed high levels of anxiety in the elevated plus-maze test with reduced open-arm exploration time (50%) relative to the control. The pomegranate administration prevented this state. Furthermore, in those animals that received pomegranates, normalization of lipids and glucose levels, an insulin resistance reduction, and a decrease in body weight gain were observed [7].

Other groups explored the anxiolytic effect of pomegranate extract in different animal models, where the neuroprotective activity was notable. For example, Gadouche et al. [85] investigated the neurobehavioral and neuroprotective effect of a methanolic extract of pomegranate (500 mg/kg) in male mice treated with lead (1000 ppm) for twelve weeks. The authors found that lead accumulation promoted decreased body weight and anxiety by increasing time in dark sites by 83%, as measured by the black/white box test. At the same time, the concomitant administration of pomegranate peel extract prevented these effects, yielding values similar to the control group. Lead accumulation and cell damage in the hippocampus and frontal cortex were prevented in 90% compared with the control group [85]. On the other hand, male and female mice exposed to proton radiation showed little anxiety in the elevated zero mazes when receiving 0.6 mg/kg of pomegranates [100]. Moreover, pomegranate seed extract (4000 mg/kg) partially reduced the ischemia-induced anxiety in male rats (52% of anxiety in ischemic rats versus 28% in ischemic rats treated with pomegranates [84]. The authors proposed that the antioxidant activity of pomegranate active compounds could be responsible for the positive neuroprotective and neurobehavioral activities [85,100].

### Mechanism of Action on Specific Receptors

The anxiolytic-like activities of pomegranates and some of its components are related to more potent antioxidant activity in the brain and hepatic tissue, working specifically on enzymatic systems [85,101]. Little has been explored on neurotransmitters regarding dopamine, serotonin, and acetylcholine [101]. It is important to note that none of these reports performed specific pharmacological screenings that could allow the inference of possible mechanisms of action of pomegranates related to the anxiolytic-like response.

Flores-Bazan et al. [102] presented an extensive and integrative review of pomegranates and their compounds as an anxiolytic [102]. They reported in a narrative review some of the proposed mechanisms for different active compounds that had been individually tested and could be present in pomegranates. Briefly, the explored mechanism suggests the action of salicylic acid on the enzyme glutamic acid decarboxylase (GAD1), favoring GABAergic activity; ferulic acid on the mRNA expression of NMDA-GluN2B receptors in the hippocampus; genistein at 5-HT1A receptors; naringin, quercetin, rutin, and gallic acid with potent antioxidant activity reviewed in [102]. However, it is essential to consider that the composition of a pomegranate varies depending on the altitude, humidity, heat, and growing conditions of the fruit. Therefore, these compounds are not necessarily present in pomegranates in the same quantity required to exert anxiolytic action. Thus, by analyzing the complete extract of pomegranate, favorable actions on anxiety or other actions could result.

As mentioned before, the administration of the AE-PG prevented CAF-induced anxiety. The authors also reported that pomegranates prevented the increase in insulin and insulin resistance and regulated lipid metabolism while canceling the reduction in mRNA expression of peroxisome proliferator-activated receptor gamma (PPARγ) in hepatic tissue induced by CAF. PPAR is a therapeutic target of thiazolidinediones (glitazones) for the treatment of diabetes [103], and pomegranates have an affinity for this receptor and PPARα [104]. Indeed, some properties of pomegranates, such as weight control, regulation of lipid and glucose levels, and insulin resistance reduction, seem to be linked to the activation of PPARγ [104,105]. In the last decade, the antidepressant and anxiolytic-like effect of glitazones has been reported [106,107,108], and the expression of the inactive form of PPARγ in the amygdala was observed in response to CAF (an increase in 300% vs. control) at the same time as the increase in anxiety (reduction of 40% in the exploration of open arms in the plus-maze test) [7].

Interestingly, pomegranates prevented this increase in the inactive form of PPARγ while preventing anxiety. Furthermore, CAF also reduced pERK1/2 in the amygdale by 31%, an effect that pomegranates prevented since treated groups showed a reduction of 9%. This molecule regulates the phosphorylation of PPARγ (Jahrling et al. 2014, 103); therefore, pomegranates may activate PPARγ via the ERK1/2 signaling pathway in the amygdala to regulate anxiety-like behavior [7].

## 5. Effects on Oxidative Stress Related to Anxiolytic- and Antidepressant-Like Effects

Another mechanism proposed to explain the effect of pomegranates involves its antioxidant properties. Evidence shows that depending on the origin of the fruit extract, different effects on lipid peroxidation (superoxide dismutase, malondialdehyde, and glutathione peroxidase activity) can be observed, with flax seed being stronger versus pomegranate peel [73]. Similarly, Cervantes-Anaya [4] reported potent antioxidant activity (formation of reactive oxygen species, lipid peroxidation, and malondialdehyde as indices of cell viability) of pomegranate extract enriched in ellagitannins in the liver and brain [4]. The author also evaluated the activity of two active compounds that could be involved in the antidepressant-like activity of the AE-PG, punicalagin and ellagic acid. Both compounds exerted antidepressant-like action at doses in the range of 0.01 and 0.1 mg/kg (i.p) on the FST; both decreased ROS formation but had different activity on lipid peroxidation and malondialdehyde, with ellagic acid being more potent than punicalagin [4]. In addition, both compounds’ scavenger activity was evaluated compared to the AE-PG’s activity. Pomegranate exerted a potent activity against superoxide and hydroxyl radicals but less on peroxynitrite since the IC50 (inhibitory concentration in vitro to reduce the number of reactive species by 50%) for each free radical was 0.012 ± 0.001 µg/mL, 0.361 ± 0.0073 µg/mL, and 1.334 ± 0.21 µg/mL, respectively. Punicalagin was more potent toward peroxynitrite (0.247 ± 0.02 µg/mL, IC50), superoxide (0.015 ± 0.002 µg/mL, IC50), and hydroxyl (0.422 ± 0.024 µg/mL, IC50), unlike ellagic acid, which lacked activity on peroxynitrite and hydroxyl and showed less activity on superoxide (0.498 ± 0.004 µg/mL, IC50) [4]. It is important to mention that the compounds evaluated crossed the blood–brain barrier, exerting a strong antioxidant effect. This information opens the opportunity for exploring how different active compounds could be isolated depending on the region where the extract comes from, having different effects in reducing stress-induced adverse events.

Similar protection was reported in anxiety models. For example, when mice challenged with AlCl3 were used, an increase in oxidative stress, as well as anxiety- and depressive-like behaviors, were detected in different models. The administration of 20% pomegranate prevented the development of anxiety- and depressive-like behaviors, increasing enzymatic oxidative biomarkers, such as catalase (CAT), superoxide dismutase (SOD), and glutathione S-transferase (GST), and the levels of non-enzymatic oxidative stress biomarkers like glutathione (GSH) [101].

It is relevant to mention two points here: (1) The specific activity of pomegranate extract on enzymatic activity biomarkers reinforces the notion of more potent antioxidant activity that could exert neuroprotective actions. (2) Anxiolytic- and antidepressant-like properties of pomegranates are conspicuous under stress conditions, and the main effect seems to restore normal function. This assumption comes from Abu-Taweel and Al-Mutary [101], who reported that in those animals that were not challenged with AlCl_3_, pomegranates did not induce any effect (light/dark test) and, indeed, an anxiogenic effect was observed (elevated plus-maze).

Consistent with this idea, Riaz and Khan [109] reported that the juice of pomegranates in unstressed male rats after 15 days of treatment produced a marginal effect on anxiety [110]. However, a combination of *Citrus limon* juice with pomegranates was evaluated in the elevated plus-maze test in unstressed male rats with an anxiolytic-like action [109]. Notably, both compounds showed positive behavioral effects on anxiety [111]. In this study, it was impossible to rule out whether the effect was mainly due to pomegranates or *C. limon*. Together, these results suggest that pomegranates exert strong anxiolytic-like action when a stressor triggers the state of anxiety. In contrast, a slight effect was observed when a stressful situation was missing, suggesting that specific HPA tone activation must be present.

## 6. Effects on Pain as a Comorbidity of Anxiety and Depression

It has been found that a high proportion of depressive disorders can be accompanied by anxiety [112] and also by chronic painful manifestations, where pathophysiological and neurophysiological similarities have been found, declaring that depression–anxiety–chronic pain comorbidity requires a search for effective therapeutic interventions [113]. Through preclinical studies and local or systemic administration, anti-nociceptive and anti-inflammatory activities of pomegranates have been observed using not only the juice [114] but also individual parts of the plant such as the flowers [115], leaves, fruit peel [116], or the seeds [114,117]. A polar extract of pomegranate rich in ellagitannins produced a significant anti-nociceptive response at central and peripheral levels using the formalin test in rats [8]. Alfa or beta punicalagin and ellagic acid were the most important constituents reported at a concentration of 15.77 vs. 10.30 mg per gram of pomegranate extract, which were considered partially responsible for the anti-nociceptive activity in the neurogenic and inflammatory stages of the test [8,118]. Urolithins, as previously mentioned, are the metabolites produced after consumption of pomegranates. Ellagitannins transformed by gut microbiota have also been described as anti-inflammatory agents [119,120].

The content of the constituents in a pomegranate can be different depending on the crop, geographical regions, maturity, and processing method. Pomegranates or their polar extracts from various sources have demonstrated significant anti-nociceptive and anti-inflammatory activities [121].

### Mechanism of Action on Specific Receptors

Since inflammation plays an essential role in the pathogenesis of anxiety and depression, the effects of pomegranates can impact not only pain relief but also mental disorders due to anti-inflammatory mechanisms of action improving the quality of life of people suffering from anxiety and depression individually or comorbidly with pain.

PPARγ is a member of PPARs, a group of nuclear hormone transcription factors whose presence at key sites involved in pain procession, such as the spinal cord, has been demonstrated as the primary modulator, increasing the expression of anti-inflammatory cytokines (IL-10) and reducing pro-inflammatory cytokines [122]. The nuclear factor-2 erythroid-related factor-2 (Nrf2)acts as an antioxidant regulator and inducible endogenous defense systems in the brain to counteract oxidative stress and modulate inflammatory responses coordinated with the nuclear factor-kappa light chain enhancer of B cells (NF-κB), as a transcription factor involved in the production of pro-inflammatory cytokines maintains healthy cells. However, this regulation is perturbed during pathological conditions, offering an opportunity for therapeutic intervention where natural products can be essential [123]. Pomegranate contains several bioactive compounds that have been investigated in several CNS diseases where neuroinflammation is a significant causal factor. In vitro and in vivo studies have shown potential preventive effects of punicalin, a polyphenolic component of pomegranates, on lipopolysaccharide (LPS)-induced memory deficiency and anxiety- and depression-like behaviors. This polyphenol improved memory impairment and anxiety- and depression-like behaviors caused by LPS and reduced the expression of inflammatory proteins such as iNOS, COX-2, IL-1β, IL-2, IL-6, and TNF-α. In addition, it also suppressed the expression of TLR4, IRAK4, TRAF6, IKK-β, NF-κB, p65, and HMGB1 in LPS-treated mouse brains and cultured microglial BV-2 cells [29]. In vitro studies have demonstrated that the ellagitannin punicalagin, another type of phenolic compound, has effective protective defenses against TNF-α/IFN-γ-induced skin inflammation by enhancing SIRT1 to mediate the STAT3 and Nrf2/HO-1 signaling pathways [124]. Ellagic acid significantly inhibited the NF-κB pathway and, consequently, the TNF-α, IL-1β, and IL-6 levels. It is recognized that different molecular pathways modulate its antioxidant and anti-inflammatory properties synergistically when combined with other main constituents of pomegranates, such as punicic acid [125]. Punicic acid in pomegranate seeds has been found as a potential metabolite for reversing obesity-related hyperlipidemia and non-alcoholic fatty liver disease where the activation of the TLR4/MyD88/NF-κB and TLR4/IL-22/STAT3 signaling pathways were involved, considering that this compound is an effective nutraceutical ingredient for attenuating lipid metabolic disorders [29]. These results support the information found on the normalization of lipids, glucose, and insulin resistance reduction, as well as the decrease in body weight gain observed by Estrada-Camarena et al. [7].

**Figure 3:**
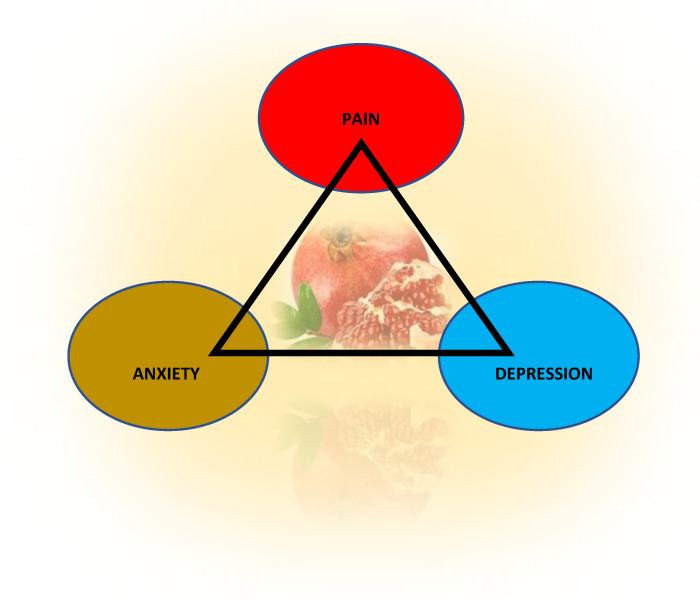
Comorbidity among depression, anxiety, and pain. These pathologies are the target of different active compounds from pomegranates, suggesting that their regular consumption could contribute to decreasing their symptomatology and improving these health conditions.

A preliminary search in the PubMed database on scientific research on pomegranates and pain or inflammation, anxiety, or depression allowed us to identify that the interest in this natural product has been evident since 2004, associated with antidepressant effect and, four years later, with anxiety. Information on pain or inflammation has been the most explored, with 44 results. In comparison, only two results described the combination of depression and pain or inflammation, and none described anxiety and pain or inflammation together (Figure 3). However, pomegranates could be a comprehensive therapy for one, two, or three of these conditions, as similarities in pathophysiology and neurophysiology, as well as mechanisms of action, may be involved.

## 7. Is It Possible to Have a Common Mechanism of Action?

The etiology of affective disorders is complex, and several hypotheses from different perspectives have been proposed. In relation to depression and anxiety, besides psychosocial and environmental factors that act as stressors, epigenetic factors that respond to environmental changes and polymorphism in specific genes related to neurotransmitter systems have been involved. Stressors activate the hypothalamus–pituitary–adrenal (HPA) axis, which, in turn, releases glucocorticoids (cortisol and corticosterone) to initiate the negative feedback in several brain areas such as the amygdala, hippocampus, and hypothalamus via glucocorticoid receptors to stop its activation. In parallel, glucocorticoids activate the sympathetic nervous system to promote the release of catecholamines (norepinephrine and epinephrine) to mobilize energetic sources to cope with physical and environmental stressors [126]. Under chronic or short-term exposure to high-intensity stress, the HPA axis regulation could be damaged due to the down-regulation of its receptors by exposure to high levels of glucocorticoids. This condition, combined with continued exposure to stressors and vulnerability, may predispose it to alter the immune system with the simultaneous release of pro-inflammatory cytokines, which in turn may activate the production of reactive oxidative species and, together, may eventually reach the brain and promote a neuro-inflammatory state [127,128].

Neuroinflammation is related to depression, anxiety, and other neuropsychiatric disorders, in which an imbalance in serotonergic, dopaminergic, GABAergic, and glutamatergic, among other neurotransmitter systems, has been reported [128,129]. Many antidepressant therapies increase the functionality of these neurotransmitters by activating several intracellular pathways discussed elsewhere [127]. Notably, through several mechanisms, many treatments promote the increase in neurotrophins, such as brain-derived neurotrophic factor, which increases neuroplasticity and synaptogenesis [130].

In addition, alterations in HPA axis function have been identified in fatigue syndrome and pain disorders such as fibromyalgia, obesity, and metabolic alterations, such as diabetes mellitus type II and metabolic syndrome [126], which are comorbid with anxiety and depression [112]. So, it might be beneficial to search for treatments that exert comprehensive effects on chronic diseases and mood disorders without relevant adverse events.

Consumption of 500 mL of pomegranate juice (1685 g/L of polyphenols) for four weeks reduced urinary cortisol and cortisone in healthy subjects. However, no significant effects were detected when compared against the placebo group. Interestingly, in this protocol, pomegranates also reduced insulin, HOMA-IR, and blood pressure [59].

Other studies evaluating menopause symptoms in terms of hot flashes, quality of life, and sleep disorders showed positive effects [49]. It is important to mention that pomegranates are frequently combined with other phytonutrients and herbs, making it challenging to identify the actual effect on the HPA axis. Additional basic research also evaluated corticosterone levels in response to pomegranate extracts, and the results are controversial. Some reports with chronic stress protocols applied reported that the consumption of pomegranate extract reduced and prevented stress-induced corticosterone increase [73,101], while after acute intense stress, no effect was observed [131]. The present data suggest that the action of pomegranates on the HPA axis activity may depend on the type of stressor.

**Figure 4:**
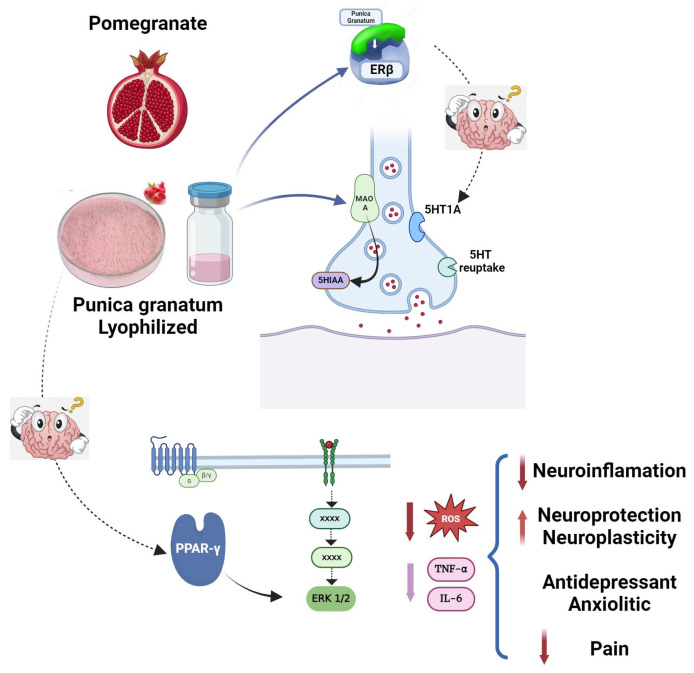
A common mechanism of action for antidepressant, anxiolytic, and anti-inflammatory actions of the aqueous pomegranate extract (*Punicagranatum*). The bioactive compounds contained in pomegranate extract could increase the activity of monoamine oxidase type A (MAO-A) enzyme in serotonergic neurons, facilitating the levels of 5-hydroxi-indolacetic acid, which, in turn, activates the peroxisome proliferator-activated receptor gamma (PPARγ). Activation of this receptor in specific brain areas may contribute, via ERK1/2phosphorylation, to decreasing anxiety and reducing oxidative stress by decreasing reactive oxygen species formation (ROS). This could also impact the decrease in pro-inflammatory cytokines, which may influence inflammation and pain. In addition, pomegranate components can also activate estrogenic receptor type β, favoring the activity of the serotonergic system to exert antidepressant-like action and contribute to the activation of PPARγ.

From the current results, it can be observed that pomegranates showed activity in the serotonergic system and ERβ. It also prevented the increase in inactive forms of PPAR in the nuclei of the amygdala and showed potent antioxidant activity. All these targets are involved in the regulation of anxiety- and depressive-like behaviors. Could these targets be related? One possibility (Figure 4) is that PPARγ activation is initiated by an increase in serotonin, which MAO-A could metabolize to produce 5-HIAA, which activates PPARγ. Consequently, this could decrease the oxidative stress (lipid peroxidation, nitrosamine formation, etc.) induced by different stressors, reducing pro-inflammatory cytokines such as IL-6 and TNF-α and microglia activation [131]. In vitro evidence showed the serotonin metabolites activate PPARγ via MAO-A [132], and the effect of pomegranates on the FST indicated that serotonin is necessary to induce an antidepressant-like action [12]. In addition, the participation of MAO-A was demonstrated by the action of pomegranates in the chronic mild stress model [73], while the involvement of PPARγ in preventing CAF-anxiety behavior [7] was also reported. Furthermore, the activity of pomegranates as an antioxidant and antidepressant was demonstrated [4], and the relationship between PPARγ and estrogen receptors was explored with in vitro studies, evaluating apoptosis in neuronal cells [133] and adipocytes that regulate lipid accumulation [134]. Thus, the results imply this is plausible, and future studies could provide evidence to support this assumption.

Some relevant symptoms of menopause due to estrogen deficiency are the metabolic alterations of lipids and glucose, which increase the risk of cardiovascular pathologies, weight gain, and risk of osteoporosis [98]. All these symptoms related to psychiatric disorders could negatively impact the quality of life of women in transit to menopause. The consumption of pomegranates could represent an alternative to alleviate or control some symptoms and complement some pharmacological therapies.

A non-exclusive explanation arises from the concept of adaptogens. Adaptogens are initially described as “metabolic regulators that increase the ability of an organism to adapt to environmental factors and to avoid damage caused by such factors” [135]. However, the concept is more complex because these types of compounds, mainly phytochemicals, exhibit multitarget activities in different receptors, such as corticosteroid, mineralocorticoid, progestin, estrogen, serotonin, N-methyl-D-aspartate, nicotinic acetylcholine, receptor tyrosine kinases, and many G protein-coupled receptors (reviewed in [136]), among others. In most of them, their activity is related to stress hormones and key mediators of the regulation of homeostasis.

Panossian [136] proposed that adaptogens are explicitly related to stress-protective activity and increased organism adaptability. Consequently, adaptogens exhibit multivalent beneficial effects against chronic inflammation, atherosclerosis, neurodegenerative cognitive impairment, metabolic disorders, cancer, and other aging-related diseases. From this point of view, pomegranates and their phytochemicals are excellent candidates to be considered as adaptogens since they restore lipid metabolism, glucose, and insulin resistance [7]. They also produce anxiolytic- and antidepressant-like actions [7,11,12], regulate oxidative stress [4], and can alleviate pain and inflammation [8].

## 8. Concluding Remarks

Mental health is relevant to human well-being. Mental disorders are frequently comorbid with chronic diseases such as obesity or diabetes, which may contribute to the development of cardiovascular problems. In some cases, a common denominator of these conditions is the chronic inflammatory process. For these reasons, it is relevant to discover alternative treatments that reduce adverse events.

The AE-PG showed regulatory properties of the HPA axis and serotonergic system, which, together with the activation of estrogen receptors, produced antidepressant-like action; in addition, the activation of PPARγ is related to anxiolytic and metabolic effects, decreasing oxidative stress and inflammatory processes. Furthermore, the high polyphenol content in pomegranates confers regulatory properties for multiple targets. These properties suggest that pomegranate extracts could be used as an adjuvant treatment for chronic diseases comorbid with anxiety and depression, and due to their high content of phytoestrogens, they can also be used to relieve menopause symptoms in which a comorbidity state could be present.

A limitation of this study could be that this revision includes only the pharmacological effects of *Punica granatum* for specific psychiatric disorders, anxiety, and depression, as well as pain, because they are the most prevalent in the general population. Nevertheless, it ought to be interesting to explore in the future the impact of this natural product on other types of psychiatric disorders since it can generate neuroplasticity and influence several changes and actions in the brain.

Another limitation is the fact that this is a narrative review. However, the limited number of available papers points out the need to work on the effect of pomegranate intake on mood disorders, stress perception, and interactions with psychotropic drugs.

## 9. Future Directions

The current data provide evidence for a possible mechanism of action underlying the anxiolytic, antidepressant, and anti-inflammatory effects of pomegranate extract that requires demonstration. In addition, it is relevant to evaluate whether interactions with other drugs used for cardiopathy, diabetes, dyslipidemia, and hormones have positive or negative effects due to the high content of polyphenols that could inhibit several forms of the enzymatic complex CYP450, particularly when polypharmacy is present.

## Figures and Tables

**Table 2:** Effects of pomegranate consumption on menopause symptoms, anxiety, depression, or stress perception.

Treatment	Dose	Sample	Outcomes	Type of Study	Effect	Reference
**Pomegranate formulation**	77.82 mg/12 w	100 (51, 49), from 45 to 60 y, healthy women	Menopause rating scale	RCT	Improved vegetative symptoms vs. placebo group. Overall, MRS was not significant.	[55]
**Pomegranate formulation**	500 mg/twice a day/8 w	78 from 45 to 65 y, healthy women	Menopause rating scale	Pre–post	Sleep disturbances and hot flashes decreased. Other symptoms were not reported individually.	[57]
**Pomegranate formulation**	500 mg/twice a day/4 w	44, from 40 to 65 y, healthy women	Menopause rating scale	Pre–post	Sleep disturbances and hot flashes decreased.	[58]
**Pomegranate juice**	500 mL/once a day/4 w	28, from 40 to 65 y, healthy women	Glucocorticoid levels, insulin, and blood pressure	Pre–post	Decreased ratio of cortisol/cortisone urine and saliva, insulin, and blood pressure.	[59]
**OCTA^®^ *W. somnifera,*** ***Lagerstroemiaspeciosa, Bacopamonniera, Zizyphusjujuba,*** ***Morindacitrifolia, Punicagranatum, Shisandraechinensis*** **and *Lyciumbarbarum*.**	30 mL/once a day/3 months	17 (10 females), healthy participants	Quality of life, perceived stress, state/trait anxiety, and depression	Pre–post	Improvement in all scales from 40 to 50%.	[60]
**Isoflavane pomegranate**	*Aframomummelegueta* (100 mg) + *Punicagranatum* (100 mg)/250 mg/once a day/8 w	34 (17–17) from 45 to 65 y, healthy women	Global health related to the quality-of-life score according to the Cervantes scale	RCT	Global quality of life, according to the Cervantes Scale, improved. No significant effect on psychic domains.	[61]
**Pomegranate pills**	1 g/once a day/2 w	11 (6–5), 2 females), 58 y, post-stroke participants	Mini-Mental State Examination, Repeatable Battery for the Assessment of Neuropsychological Status	CT	Neuropsychological status improved, state and trait anxiety (10%), Beck score decreased, and self-care increased (15%).	[62]

Data from the literature using extracts of pulp, juice, peel, or seed of pomegranates. RCT = randomized controlled trail; CT = controlled trial.

## Data Availability

No new data were created or analyzed in this study. Data sharing does not apply to this article.
